# Search and insight processes in card sorting games

**DOI:** 10.3389/fpsyg.2023.1118976

**Published:** 2023-05-05

**Authors:** Michael Öllinger, Eörs Szathmáry, Anna Fedor

**Affiliations:** ^1^Parmenides Center for the Study of Thinking, Pöcking, Germany; ^2^Psychological Department, Ludwig-Maximilians-University of Munich, Munich, Germany; ^3^Parmenides Center for the Conceptual Foundations of Science, Pöcking, Germany; ^4^Institute of Evolution, Centre for Ecological Research, Budapest, Hungary

**Keywords:** insight, rule learning, problem solving, restructuring, AHA, search, variation

## Abstract

Insight problems are particularly interesting, because problems which require restructuring allow researchers to investigate the underpinnings of the Aha-experience, creativity and out of the box thinking. There is a need for new insight tasks to probe and extend the limits of existing theories and cognitive frameworks. To shed more light on this fascinating issue, we addressed the question: Is it possible to convey a well-known card sorting game into an insight task? We introduced different conditions and tested them via two online experiments (*N* = 546). Between the conditions we systematically varied the available perceptual features, and the existence of non-obvious rules. We found that our card sorting game elicited insight experience. In the first experiment, our data revealed that solution strategies and insight experience varied by the availability and saliency of perceptual features. The discovery of a non-obvious rule, which is not hinted at by perceptual features, was most difficult. With our new paradigm, we were able to construe ambiguous problems which allowed participants to find more than one solution strategy. Interestingly, we realized interindividual preferences for different strategies. The same problem drove strategies which either relied on feature integration or on more deliberate strategies. The second experiment varied the degree of independence of a sorting rule from the standard rules which were in accordance with prior knowledge. It was shown that the more independent the hidden rule was, the more difficult the task became. In sum, we demonstrated a new insight task which extended the available task domains and shed light on sequential and multi-step rule learning problems. Finally, we provided a first sketch of a cognitive model that should help to integrate the data within the existing literature on cognitive models and speculated about the generalizability of the interplay of prior knowledge modification and variation for problem solving.

## Introduction

### Insight

Let's try to solve the following problem (light-switches-problem, unknown source). Imagine we have three light switches outside of a house and one light bulb inside. We cannot directly see the light from the outside. The front door is closed. What we know is that the light is switched off at the beginning. We are allowed to play around with the three switches as we like. However, if we plan to open the door we have to leave the switches as they are. How can we find out for sure which switch controls the light bulb?

After fiddling around for a while, we might realize that the problem cannot be solved easily. The given information is not directly providing a solution. To solve the riddle we need to integrate information that is not obvious. Eventually, we might remember from our prior knowledge that light bulbs become warm when current flows through them. Now, we realize that we can use temperature as an additional source of information that helps us to find the solution. Such an insight in the solution of a difficult problem could be accompanied by an Aha-experience. We leave it to the reader to figure out the solution.

In general, it has been assumed that insight problems force us to restructure the given problem representation. The scientific domain of insight problem solving has been dealing with the question: How do problem solvers find a solution to a difficult problem which cannot be solved by using prior knowledge or standard procedures, but which needs restructuring? (Ohlsson, 1992; Dominowski and Dallob, 1995; Cunningham et al., 2009).

Öllinger and Knoblich (2009) provided three different perspectives on the concept of insight.

*First*, from the *phenomenological perspective* the Aha-experience demarcates solving a problem with insight or without insight. Until now most studies relied on the subjective Aha-experience to differentiate insight problem solving from non-insight problem solving. For neuroscientific studies this assumption had great importance to contrast the brain activities, recorded with MRI or EEG, during the solution of problems with or without an Aha-experience (Jung-Beeman et al., 2004; Luo et al., 2004; Mai et al., 2004; Bowden et al., 2005; Sandkühler and Bhattacharya, 2008; Kounios and Beeman, 2014). Currently, there is promising ongoing research detailing the facets of subjective experience such as confidence, pleasure, certainty, etc. (Danek et al., 2013, 2016; Webb et al., 2016, 2018, 2019; Danek and Salvi, 2020).

*Second*, the *task perspective* states that certain problems (tasks, riddles, puzzles) elicit insight with a high probability (Duncker, 1945; MacGregor et al., 2001; Öllinger et al., 2013b, 2016).

*Third*, the *process perspective* distinguishes insight from non-insight by the underlying cognitive processes. The key process was assumed to be restructuring (Wertheimer, 1959; Ohlsson, 1984a,b; Dominowski and Dallob, 1995; Öllinger et al., 2014). Additionally, the process perspective also considered the temporal course of insight problem solving and was often characterized by a non-stepwise process in contrast to a stepwise (gradual) process for analytical problem solving (Metcalfe, 1986, 1998; Zander et al., 2016).

For our purpose, we utilized all three perspectives. We assumed restructuring to be the key concept which demarcated insight from non-insight tasks. In order to measure restructuring we relied on the subjective Aha-experience of our participants. Finally, we aimed to develop a new insight task domain which can be used to run new behavioral and neuroscientific insight experiments.

In the next paragraph, we detailed the key concept of restructuring, which provided the basic cognitive process.

### Restructuring and representational change

There has been agreement that insight problem solving is tightly linked to the process of restructuring (Weisberg, 1995; Bowden et al., 2005; Cunningham et al., 2009; Kounios and Beeman, 2014; Webb et al., 2016). At the beginning, the Gestaltists explained restructuring according to perceptual laws (Köhler, 1925; Wertheimer, 1925, 1959; Koffka, 1935; Duncker, 1945). Later, Wertheimer (1959) developed a general theory of human thinking unifying the fields of perception, thinking, and social interactions—restructuring was assumed to be the overarching cognitive faculty.

Ohlsson (1984a,b) transferred the Gestaltists' notion of restructuring into the cognitive term of representational change. The representational change theory (RCT, Ohlsson, 1992, 2011; Knoblich et al., 1999) was able to provide clear and testable assumptions. RCT was understood as an extension of the problem space theory (Newell et al., 1958; Newell and Simon, 1972), which proposed that problem solving had to be seen as a search through a given problem state space.

RCT relied on two main assumptions:

- First, perception affects *problem representation*, e.g., chunking of the problem elements. Chunk decomposition is the mechanism which breaks chunks into pieces and builds new chunks.
- Second, prior knowledge induces self-imposed constraints on the *goal representation*. The goal representation determines the task set. That is, prior knowledge activates the set of rules which have to be obeyed to solve the problem (Frith, 2000; Reverberi et al., 2005; Chi and Snyder, 2011). As a consequence the search space is constrained by the rules. Constraint relaxation helps to overcome such constraints and results in more flexible representations, which allow it to apply other rules (Knoblich et al., 1999; Öllinger et al., 2013a,b; Danek et al., 2014).

The basic idea of this approach can be nicely illustrated by the matchstick arithmetic tasks (Knoblich et al., 1999). The authors started with the theoretical predictions of the RCT and searched for problems that met its predictions. In matchstick arithmetic tasks one had to change an incorrect arithmetic statement into a correct one by moving a single matchstick. The statements were written in Roman numbers, composed of matchsticks. According to RCT, tasks in which values had to be changed (e.g., VI = VI + II -> VI = IV + II) should be easier than tasks in which operators had to be changed (e.g., VI – VII = I -> VI = VII – I), because the latter needed a goal representation where operators were represented as variable. The most difficult problems should be those in which the common structure of the equation had to be changed (e.g., VI = VI + VI -> VI = VI = VI). Here operators were represented as variable and at the same time the structure of the equations had to be changed so that a tautology resulted. These predictions were exactly confirmed by empirical data. In our understanding, this means that the more flexible the rules were the more difficult it was to find those rules.

Another line of research which built on the problem space theory (Kaplan and Simon, 1990; MacGregor et al., 2001; Ormerod et al., 2002) was focusing on the search for and the application of appropriate heuristics, which helped to navigate the search space (Newell and Simon, 1972) in a more deliberate way. In this understanding, restructuring follows regularly when the appropriate heuristics are used (e.g., a hill-climbing or progress-monitoring heuristics). This account emphasized a deliberate search for promising states and rules which could solve insight problems.

### Card sorting and insight

In this paragraph, we collect arguments for a new insight task, which relied on finding non-obvious rules by representational change in a card sorting game. Generally, there was a broad variety of insight tasks ranging from verbal riddles or puzzles to geometric problems and mathematical problems (Weisberg, 1995; Knoblich et al., 1999; Dow and Mayer, 2004; Jung-Beeman et al., 2004; Kershaw and Ohlsson, 2004; MacGregor and Cunningham, 2008; Öllinger et al., 2013a,b). Recently, even magic tricks were successfully utilized to investigate insight problem solving (Danek et al., 2014). Mostly, the applied problems provided an initial representation that needed the realization or manipulation of particular pieces of information to overcome self-imposed constraints. To give an example, the frequently used remote associates tasks (RAT, Jung-Beeman et al., 2004; Webb et al., 2018) required participants to find a compound word given three words (e.g., crab, sauce, pin; the solution word is apple). The solution was either correct or incorrect and had to be found very fast. The solution process remained widely opaque. Consequently, with these problems it will be difficult to trace the steps of the problem solving process. Moreover, many of the existing tasks relied on semantic and prior knowledge about a domain (e.g., arithmetic knowledge in the matchstick tasks (Knoblich et al., 1999), or prior knowledge constraints in magic tricks (Danek et al., 2013, 2014), which then was corrupted by certain tasks (e.g., see the tautological equation above). All these aspects limited, at least in our understanding, the amount of data that could be gathered during the problem solving process.

We see a number of advantages in the use of card sorting games to investigate new facets of insight problem solving. Card sorting games rely on simple features such as color, number or shape. Playing with these stimuli requires almost no prior knowledge or expertise. The rules are mostly obvious and the application of the rules can be properly monitored in a trial-based manner. The number of trials and the switching of rules can be set and freely varied.

These convenient sets of properties might enable researchers to introduce new and not obvious features. Here we see the potential to convey card sorting games into insight-type problems following the assumptions of the RCT. Another problem that some classical problems entailed, was the fact that it could be unclear whether the solution actually required representational change or was accomplished in a deliberate way. We assume that card sorting games inherently have the potential to disentangle insight from deliberate thinking processes by clearly pinpointing at which stage of the process a representational change was necessary. Card sorting games allow us to create sets of cards that can be ambiguous or unambiguous. That is, each card can either go to a certain target card or there is more than one target card which offers a mapping criterion so it is more or less obvious which rules have to be applied.

Finally, we also see the potential that the sequential aspect of card sorting games can further inform us about the interplay of incidental learning processes and restructuring. Haider et al. proposed and demonstrated with the Number Reduction Task (Haider and Frensch, 1996; Wagner et al., 2004; Haider and Rose, 2007) that incidental learning principles, sequence learning, and changes in performance could result in representational changes and insight-type experiences. Moreover, sequential problem types may help to scrutinize participants' hypothesis updating process and might bridge the gap between insight problem solving and Bayesian inference (Griffiths et al., 2008).

### The current study

The main goal of our study was to apply the assumption of the RCT to a newly developed card sorting game in order to create an insight-type task domain.

**Figure 1:**
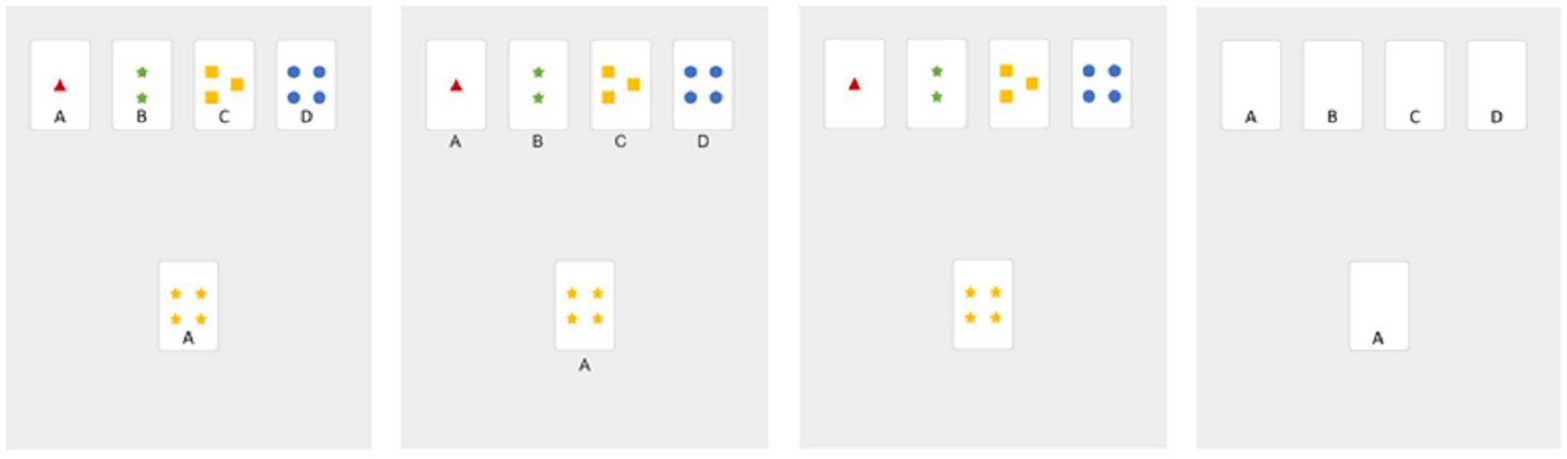
Screenshots from Experiment 1. Conditions from left to right: Letters On, Letters Below, No Letters, Letters Only. The correct key card is the leftmost key card in all conditions.

We aimed at providing a proof of concept that the alleged potentials of card sorting games for insight were justified. This endeavor was intended to support researchers to investigate the search and hypothesis testing processes in more detail and to distinguish deliberate and implicit processes. We copied the inner logic of the light-switches-problem (see introduction) which asked to find a hidden and not obvious property or rule to relax the initial problem representation. We identified the classical Wisconsin Card Sorting Test (WCST, Heaton et al., 1993) as an appropriate candidate for our purpose. The classical version of the WCST was mostly used for neuropsychological assessments. Usually, patients were asked to sort a deck of cards (source cards) onto four target cards (or key cards; see Figure 1, column three), without knowing the sorting rule. The cards differed in three dimensions: the color of figures, the number of figures and the shape of figures on the face of each card. Each dimension had four possible features or values: color (red, green, yellow, blue), number (one, two, three, four) and shape (triangle, star, square, circle).

Consequently, the cards could be sorted by a color rule, a number rule and a shape rule (*standard rules*), where the correct target card is the one that matches the source card in the given dimension. While sorting the cards, participants were receiving either “correct” or “incorrect” feedback after each move. After a predefined number of trials the sorting rule was switched.

We modified the classic WCST by introducing new sorting rules (see later). In two experiments, we introduced new rules ranging from simple feature matching to the application of more complicated rules which relied on non-obvious or hidden dimensions.

We were interested in whether these manipulations changed the problem difficulty (solution rate), solution time, and the Aha-experiences according to the assumptions of the RCT. We were also interested in answering the question which rules participants selected under conditions where a deliberate or an insight-type solution was available.

## Experiment 1

The task in each condition was to find the underlying sorting rule that matched the deck cards to the target cards. We introduced three different conditions and one control condition. The problem difficulty was varied by the availability of obvious to non-obvious rules. In the control condition there was only one exclusive mapping criterion. The problems were designed in a way that we could compare simple feature mapping with the more difficult integration of information and deliberate rule induction.

### Design and hypotheses

For the first experiment we introduced a new and non-obvious rule. This new rule required the problem solver to match deck cards with target cards that shared no single feature (not the same color, not the same number, not the same shape). We named this rule the *exclusion rule*. We used a deck of source cards, which unambiguously matched each target card (see Appendix for the list of source cards).

In the three conditions of Experiment 1 (see Table 1), source cards could be sorted correctly by applying the *exclusion rule*. In two of these conditions, we assessed the difficulty by providing a new rule. In these conditions, it was possible to use instead of the exclusion rule a much simpler rule, because the correct sorting rule was indicated by letters. That is, it was not necessary to infer the exclusion rule, but to realize that the letters indicated the correct target card [A (source card) goes to A (target card)]. As a consequence, a new feature based rule (matching letters) could be applied. In a further variation, we were interested in whether letters would also be realized as helpful when they were presented outside the cards. That is, the letters were not part of the cards, but first had to be integrated to become useful.

**Table 1:** Summary of Experiment 1 with the description of the deck and the possible sorting rules.

**Condition**	**Description of the deck**	**Can it be solved based on the letter rule?**	**Can it be solved based on the exclusion rule?**
Letters On	Letters within cards	Yes	Yes
Letters Below	Letters below cards	Yes	Yes
No Letters	No letters	No	Yes
Letters Only	Letters on the cards, but no figures	Yes	No

Concretely, in the *Letters On* and the *Letters Below* condition the letters A, B, C and D (source cards) indicated the correct target card (see Figure 1). In the *Letters On* condition the letters were displayed on the bottom of the cards. In this condition we assumed that the letters could be integrated as a further standard rule, it would be very unlikely to use the more difficult exclusion rule. In the *Letters Below* condition the letters were displayed below the cards. It was not obvious that the letters were part of the solution. Therefore, we expected that participants could ignore the letters and induced the exclusion rule instead. We predicted that the Letters Below condition would be more difficult, because participants had to relax the constraint that required information was only printed on the cards as it is usually in card games. Further, they had to change the problem representation and build a new chunk of the given information (chunk decomposition; Knoblich et al., 1999).

In the third condition (No Letters), participants could only rely on the exclusion rule. We assumed that constraint relaxation was necessary to overcome the application of the standard rule set. After that, a new and deliberate search for new and promising states could be initiated (MacGregor et al., 2001). We predicted that the No Letters condition was the most difficult.

To sum up, we predicted the following order of problem difficulty for the three experimental conditions: Letters On < Letters Below < No Letters. We tested these predictions by comparing solution rates and solution times between conditions in a between-subject design. A task was defined as more difficult, if fewer participants were able to solve it, or if it took longer to solve it (in the case that solution rates were the same).

In a pilot study, we realized that participants reported an unexpectedly high number of Aha-experiences even in the presumably easiest condition (Letters On). As a consequence, we introduced an Aha!-control-condition (*Letters Only*) to determine a baseline for the Aha-experiences. In this condition, all cards had exclusively a letter at the bottom, but there were no other symbols printed on the face of the source deck (see Figure 1, rightmost picture). We predicted that all three of the experimental conditions elicited a higher proportion of Aha-ratings than the Letters Only condition.

### Methods

#### Participants

We recruited participants online via the Prolific platform (www.prolific.co) for Experiment 1 and 2. Prolific is an online platform for online research. Only participants with English as their first language participated in our study. They were redirected to our website and assigned to either Experiment 1 or 2.

We excluded the data of those participants who played the game more than once, refreshed the screen during the game or went back to the instructions page after starting the game, had missing data or indicated that they were colorblind. We stopped the study when each condition had 78 participants [We used the G^*^Power 3.1 software (Faul et al., 2007) to estimate the required sample size—see Appendix for the exact settings]. Three hundred and twelve participants (78 in each condition, 167 female) were included in the data analysis of Experiment 1.

Our experiments obeyed the World Medical Association (2013). We followed the code and the ethical principles of the German Psychological Society and the European Commission.

#### Procedure

Participants were asked to read and confirm that their participation was voluntary, their data was stored anonymously and that they were compensated for their participation. At the next page, we asked for age, sex, colorblindness and handedness data. The following page provided instructions for the task: some explanation (see below) at the top of the page and a figure at the bottom, which illustrates the card sorting task (similar to Figure 1, third column).

“STOP!
Please read the rules of the game carefully.
Once you have started the game, you may not return to this site. Please do not press the back or the refresh button of your browser. If you do so, your data cannot be used.
In the following game, your task will be to find out how to sort the given cards. You will see a deck of cards at the bottom of the screen and four key cards at the top of the screen (see figure below). Please, drag the cards from the deck one-by-one and drop them onto one of the key cards.

After each move you will be informed whether you sorted the card correctly or not.”

After clicking the “Continue” button, the faces of the four target cards and the deck of source cards showed up on the screen. Participants could drag-and-drop the top card from the deck onto one of the key cards. After moving a card, participants got feedback in the form of an on-screen message, whether the move was “Correct!”, printed in blue color at the middle of the screen, or “Incorrect!” in red color. After the feedback, the source card disappeared and the next card of the deck was presented.

The game was over, if either participants correctly sorted 18 cards in a row (solver), or the upper time limit of 15 min was reached (non-solver). After the game, participants completed a post-experiment questionnaire. The questionnaire asked the following questions:

- What do you think the goal of the experiment was?^*^
- Did you find a rule for sorting the cards? If you did, describe the rule!
- Have you experienced an Aha! feeling any time during the experiment? An Aha! is characterized by suddenness and obviousness. Like an enlightenment. It accompanies an unexpected and unintended solution to a problem. You are relatively confident that your solution is correct without having to check it (Yes/No)^*^.
- How difficult did you find the experiment? (1 extremely easy-−10 extremely difficult)^*^.
- Any further comments?

The definition of an Aha! experience was an adaptation of the instructions of Danek et al. (2014). Questions marked with an asterisk (^*^) were required to finish the experiment. Each participant attended only one condition.

#### Materials

The online experiment was programmed in JavaScript and PHP. JavaScript provided the frontend interaction with the user. PHP provided the backend storage of the data on our server and the control of the experimental procedure. The experiment ran on a Linux server.

The deck consisted of 24 cards. The list of cards could be found in the Appendix. The deck was shuffled randomly in all conditions of Experiment 1. After all cards in the deck were sorted, the deck was reinitialized.

#### Data analysis

All data and analysis scripts can be found here: https://osf.io/w9sbe/?view_only=d165197f4f86448d98f00e6feb93c943. The data analysis script was written in R Markdown (R Core Team, 2013). We analyzed the contingency table containing the number of solvers and non-solvers in pairs of conditions with Fisher's exact test. A *p* < 0.05 indicates that the row/column association was statistically significant.

For the solution time analysis we used a one-way ANOVA with the between-subject factor Condition, if the data was normally distributed, or a two-sample Wilcoxon test (same as the Mann–Whitney test), if it was not. All tests are two-sided.

### Results

#### Difficulty of the task

The number of solvers (out of 78 participants per condition) was 78 (100%) in the Letters On condition, 73 (94%) in the Letters Below condition, and 50 (64%) in the No Letters condition (Table 2).

**Table 2:** Overview of solution rate and solution time across conditions.

**Condition**	**Solver**	**Non-solver**	**Solution time, *M* (min)**	***SD* (min)**
Letters On	78	0	1.60	1.77
Letters Below	73	5	3.56	3.58
No Letters	50	28	5.95	4.00
Letters Only	78	0	0.85	0.63

There was no significant difference between the Letters On and Letters Below conditions (*p* = 0.059, Fisher's exact test). There was a significant difference between the Letters Below and No Letters conditions (*p* < 0.001, Fisher's exact test).

**Figure 2:**
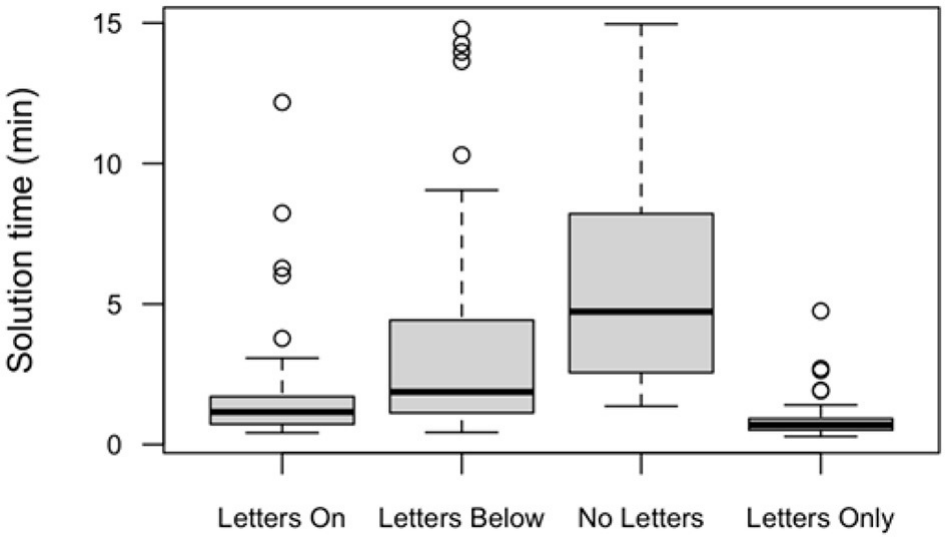
Box-plots of solution time of solvers across conditions in Experiment 1. Circles represent outliers. Whiskers extend to the most extreme data point which is no more than 1.5 times the interquartile range from the box. Please note the Letters Only condition was also used as control condition for Experiment 2.

Next, we analyzed the solution time of solvers. Solution time was defined as the time solvers spent with the card game from the appearance of the first card on the deck until the game was over. Figure 2 and Table 2 illustrated the increase of solution time and variance from the Letters On through the Letters Below to the No Letters condition. Since the data was not normally distributed, we used a two-sample Wilcoxon test to compare the solution times between the Letters On and Letters Below conditions: the difference was significant (*W* = 1,624, *p* < 0.001).

#### Aha-ratings

The Aha-rating of solvers was 58% (45/78) in the Letters Only (control) condition, 82% (64/78) in the Letters On condition, 93% (68/73) in the Letters Below condition and 98% (49/50) in the No Letters condition.

We ran pairwise comparisons to analyze the associations between condition and Aha-ratings (Fisher's exact tests). We compared all experimental conditions to the control condition (Letters Only). The analyses revealed significant results in the case of all three comparisons: Letters On *p* < 0.005, Letters Below *p* < 0.001 and No Letters *p* < 0.001. Further comparisons (Letters Below vs. Letters On and No Letters vs. Letters Below) revealed no significant differences (*ps* > 0.5), except the No Letters vs. Letters On comparison (*p* < 0.005), however if we account for multiple comparisons with a Bonferroni correction, this difference was not significant either.

#### Rule report

In the post-experimental questionnaire, participants were asked to report, if possible, a rule. M.Ö. and A.F. (raters) independently scored the answers. The raters categorized the answers as the letter rule, the exclusion rule or no rule (missing answer, unintelligible answer or ambiguous answer) for all participants. The raters went through all of the responses independently, and in the three experimental conditions of Experiment 1 (234 cases), disagreed in 13 cases (94.4% inter-rater reliability). The raters discussed the remaining cases and agreed on a category (5 of them were classified as one of the rules, the rest were classified as no rule).

For the statistical analysis we used only the data of solvers (see Table 3). We compared the letter rule and exclusion rule cell/row associations between the Letters On and Letters Below conditions with a Fisher's exact test and it revealed a significant difference (*p* < 0.001). Participants in the Letters On condition reported significantly more letter rules than participants in the Letters Below condition.

**Table 3:** Reported rules of solvers across conditions in Experiment 1 (NA, Not Applicable).

**Condition**	**Letter rule**	**Exclusion rule**	**No rule**
Letters On	69	3	6
Letters Below	44	19	10
No Letters	NA	43	7

In order to further explore the data, we analyzed solution times with two additional tests. The first test investigated whether the solution times of participants who selected the letter rule differed between the Letters On (*M* = 1.26 min, *SD* = 0.87 min) and the Letters Below condition (*M* = 2.55 min, *SD* = 2.89 min). According to the two-sample Wilcoxon test the difference was significant (*W* = 1,067, *p* < 0.01).

The second analysis tested whether there was a difference in solution time in the Letters Below condition between participants who selected the letter rule (*M* = 2.55 min, *SD* = 2.89) and participants who selected the exclusion rule (*M* = 5.18 min, *SD* = 3.95). According to the two-sample Wilcoxon test the difference was significant (*W* = 177, *p* < 0.001).

### Discussion

In Experiment 1 we varied the application of rules from an obvious feature dimension (Letters On) to the integration of additional information (Letters Below) which was not printed on the card to the induction of a non-obvious rule (No Letters). In all three conditions cards could be sorted by the exclusion rule.

Based on the RCT we predicted that it was more difficult to solve the task in the Letters Below condition than in the Letters On condition, because the integration of the letters below the cards in the search space might need representational change (chunk decomposition). The difference in solution rates was not significant between the two conditions. However, the solution times were significantly higher in the Letters Below condition. This indicated that the latter task was more time consuming. Apparently, it was more difficult to either utilize and integrate the letter information printed below the cards as part of the solution or to find the exclusion rule and to ignore the letters below (more participants reported the exclusion rule in the Letters Below condition than in the Letters On condition). Contrasting solution time of participants who either applied the letter rule or the exclusion rule revealed that the latter was significantly more time consuming. This might stress the need for a deliberate inference process to find the exclusion rule.

Finally, the No Letters condition was more difficult than the Letters Below condition: solution rates were lower. This was expected, because the No Letters condition required the exclusion rule. That is, it was crucial to overcome the initial rule set on the one hand, and on the other hand to discover a new rule which said: ignore all obvious matching criteria and move the card to the target card which met no feature.

We compared all Aha-ratings to a control condition: in the Letters Only condition the letters A to D were the only visible symbols on the faces of the cards. The Aha-ratings in the Letters Only condition were unexpectedly high (58%).

Aha-rates were higher in all three experimental conditions. The proportion of Aha-ratings increased with the difficulty of the task. Almost all solvers (98%) reported an Aha! experience in the No Letters condition. This spoke for the interplay of deliberate processes which drove a representational change (Kaplan and Simon, 1990; MacGregor et al., 2001).

Taken together, it seemed that either our provided definition of Aha! was inappropriate or subjective Aha-reports in our task differed from those found in classical insight problems. It was conceivable that Aha-experiences in our task were not necessarily linked to restructuring, but they could also be linked to surprise or to unexpected events. It is important to note that all participants saw the same instruction at the beginning. The game was introduced with images and instructions which referred to the standard rules (Figure 1, third column). Therefore, it is conceivable that the Aha-ratings were driven by the insight that the task looked different or was much easier than expected. This point needs further clarification by further empirical studies.

In general, those findings might add additional facets to the nature of Aha-ratings. The conclusion was obvious, that the phenomenological experience of Aha! differed in a multi-step card playing game from other problem types (Webb et al., 2016; Danek and Salvi, 2020).

Did we manage to create insight tasks in Experiment 1? If we relied on the Aha-ratings, then all three experimental conditions had to be classified as insight-type problems from a phenomenological perspective. From a process perspective it remained more difficult to find an answer. Particularly, the Letters Below condition provided interesting results. In this condition 19 out of 73 solvers applied the exclusion rule. That is, they did not obviously rely on the additional letter cues printed below the cards. The solution time was slower in comparison with the Letters On condition. The use of problems which could be solved by two rules provided new insights in the dynamics of feature integration vs. deliberate search for the solution. It was shown that features were used effortlessly if they were printed on the card's face.

We concluded from this evidence that the Letters Below and the No Letters conditions needed representational change, thus these tasks could be classified as insight tasks from the process perspective, too.

An interesting question is how some of the solvers did find the non-obvious rule. Was it an effortful and deliberate or an implicit and automatic process? A basic learning mechanism which allows distinguishing same from different feature combinations was investigated in animal cognition. E.g., Thomas and Frost (1983) provided stimuli that varied form (triangle, circle, square), color and size. They manipulated the number of shared features and the animals (squirrel monkeys) were trained to find the odd stimulus when confronted with three presented stimuli. The results revealed that the animals were able to learn this task, even when the combination of features was varied. Additionally, Hille et al. (2006) demonstrated for a Californian sea lion that the animal was also able to select the odd stimuli given three black printed figures on white background. In sum, these findings suggest that the categorization of same and odd stimuli might be a cross-species mechanism. Consequently, for our paradigm it is conceivable that participants relied on learning the oddity that key cards had to go to the target card with the non-overlapping features. If so, their reasoning could probably build on an evolutionary adaptive process to find the special under the same.

A final remark, when we compared our tasks with the light-switches-problem, we had to admit that there was still an important difference: Our tasks of Experiment 1 carried all the necessary information to solve the problem. Even the No Letters condition allowed the problem solvers to find the solution via combinatorial reasoning. That is, the first experiment demonstrated the easiness of manipulating features and rules in a card sorting game. In Experiment 2 we aimed at addressing this difference by introducing a hidden dimension (such as the temperature) which had to be discovered.

## Experiment 2

### Design and hypotheses

In the second experiment, we tested how properties of the deck did influence the search for a non-obvious sequence rule which added a new dimension to the search space.

In all three conditions of Experiment 2, cards could be correctly sorted by a *sequence rule* (see Table 4). Cards from the deck had to be sorted in a left to right order. Meaning, the first card in the deck should go to the leftmost target card, the second card to the second target card from the left, the third source card to the third target card from the left, the fourth source card to the rightmost target card. The fifth source card resumed the left-to-right sequence and again went to the leftmost target card, and so on.

**Table 4:** Summary of the results of Experiment 2 with the description of the deck and the possible sorting rules.

**Condition**	**Description of the deck**	**Can it be solved based on the exclusion rule?**	**Can it be solved based on the sequence rule?**
Uniform Deck	Only one type of card (the moon card)	No	Yes
Fixed Deck	Standard cards in a fixed order (excluding ambiguous moves)	Yes	Yes
Random Deck	Standard cards in random order (ambiguous moves are possible)	No	Yes

The sequence rule required participants to sort cards in a fixed order, irrespective of the information that was printed on the source or the target cards.

**Figure 3:**
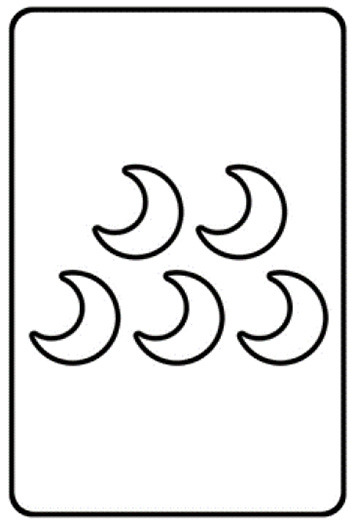
The moon card used in the Uniform Deck condition of Experiment 2. All features of the moon cards were incompatible with the standard features.

The first condition (*Uniform Deck condition*) served as a baseline condition. The deck consisted of only one single type of source card, the moon card. The moon card was newly introduced. The face of the moon card showed five white moons (see Figure 3). This card did not match any of the standard dimensions (color, number, shape of figures). Thus, we assumed that the participants overcame quickly (representational change) the standard rules and started to search for non-obvious rules.

In the second condition (*Fixed Deck condition*) the deck was presented in a way that the order excluded the possibility of ambiguous moves (see Appendix 1). That is, each card could go to exactly one target card in the defined left to right sequence. For example, the four yellow stars card could only come as the first in the sequence (see Figure 1).

Such as in Experiment 1 the Fixed Deck condition could also be solved by the exclusion rule (Experiment 1). The main difference between the exclusion rule and the sequence rule was that the first was not independent from the three standard dimensions. The sequence rule was independent from the basic dimensions. It introduced a new dimension (sequential order), which spanned a new search space, such as warmth in our light-switches-problem (see the introduction).

The Fixed Deck condition allowed us to evaluate whether problem solvers found it easier to uncover the exclusion rule or the sequence rule. This revealed whether it was easier to use the exclusion of standard features or to realize the visual-spatial order of the sequence information.

In the third condition (*Random Deck condition*), the deck was built of cards in random order. The cards could only be sorted by the sequence rule. However, in the Random Deck condition the visible features of the cards could be distracting in a way that sequential and standard rule information could interfere and be misleading (see the third screen at Figure 1). For example, if the source card (four yellow stars) showed up as the first in the sequence, then it had to be sorted to the leftmost target card. If it showed up as the second in the sequence then it had to be sorted to the second target card from the left. In the latter case, if the participant sorted the card correctly, they mistakenly concluded that shape determined the sorting criterion.

In the Random Deck condition, we assumed that participants first had to overcome the application of standard rules.

We predicted for task difficulty: Uniform Deck condition < Fixed Deck condition < Random Deck condition.

We assumed that the face of the cards was the least distracting in the Uniform Deck condition. A single card that shared no features with the key cards restricted the search space and helped to quickly find the sequence rule.

We predicted that the Fixed Deck condition was more difficult, because participants had to abstract from the visible features of the cards.

The Random Deck condition was expected to be even more difficult, because participants had to fully ignore the standard rules and detect the sequence rule.

As for the Aha-ratings, we used the same Aha!-control condition as in Experiment 1 (No Letters condition). We predicted that all three experimental conditions in Experiment 2 elicited Aha-ratings with higher probability than in the control condition.

### Methods

#### Participants and procedure

We used the same methods for recruiting and excluding participants and the same procedure as in Experiment 1. We included the data of 234 participants in the data analysis (78 per condition, 106 females). We used the data of the same participants for the Aha-control condition as in Experiment 1 (Letters Only condition).

#### Materials and design

In the Uniform Deck condition the deck consisted of only one card (see Figure 3): the moon card had five (number dimension) white (color dimension) half moons (shape dimension). It differed in all three standard dimensions from all target cards.

In the Fixed Deck and the Random Deck conditions the decks consisted of the same cards, as in Experiment 1, No Letters condition (24 of the standard cards used in the original WCST, see Appendix 1). In the Random Deck condition the same cards were randomly shuffled in the deck.

### Results

#### Difficulty of the task

Table 5 provided a summary of solution rates and solution times across conditions in Experiment 2. The Uniform Deck and Fixed Deck conditions had very similar solution rates (90 and 88%, respectively), but the mean solution time was higher in the Fixed Deck condition. The Random Deck condition had a lower solution rate (53%), but the solution time of solvers was similar to that of the Fixed Deck condition.

**Table 5:** Overview of solution rates and solution times across conditions in Experiment 2.

**Condition**	**Solver**	**Non-solver**	**Solution time, *M* (min)**	***SD* (min)**
Uniform Deck	70	8	3.53	2.35
Fixed Deck	69	9	5.58	3.74
Random Deck	41	37	5.83	3.63

We analyzed the contingency table containing the number of solvers and non-solvers with pairwise Fisher's exact tests. There was no significant difference between the Uniform Deck and the Fixed Deck conditions (*p* = 1.0). The row/column associations were highly significant for the Fixed Deck vs. Random Deck conditions (*p* < 0.001, Fisher's exact test).

**Figure 4:**
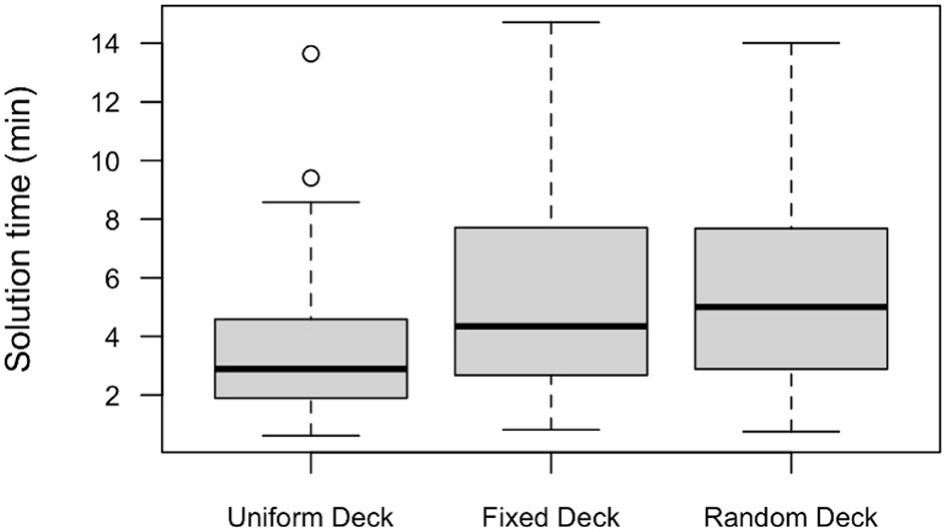
Solution time across conditions in Experiment 2. Circles represent outliers. Whiskers extend to the most extreme data point which is no more than 1.5 times the interquartile range from the box.

Figure 4 showed the boxplots of solution times for solvers across conditions. Since the data was not normally distributed, we used a two-sample Wilcoxon test to compare the solution times between the Uniform Deck and Fixed Deck conditions. The difference was significant (*W* = 1,624, *p* < 0.00).

#### Aha-rating

We used the Letters Only condition from Experiment 1 as a control for comparing the number of solvers who reported Aha-feelings during the task. The Aha-rating of solvers was 76% (53/70) in the Uniform Deck condition, 88% (61/69) in the Fixed Deck condition and 90% (37/41) in the Random Deck condition.

All three Fisher's exact tests were significant: Uniform Deck condition *p* < 0.05, Fixed Deck condition *p* < 0.001, Random Deck condition, *p* < 0.001. Comparisons between the three experimental conditions revealed no significant differences (*ps* > 0.05).

#### Rule report

We used the same rating procedure as for Experiment 1. The raters disagreed in 8 cases (8/234; 96.6% inter-rater reliability). After discussing these cases, the raters classified four of the answers as sequence rule, and four as “no rule.” In total, we found the following distribution of reported rules (see Table 6).

**Table 6:** Reported rules of solvers across conditions in Experiment 2 (NA, Not Applicable).

**Condition**	**Exclusion rule**	**Sequence rule**	**No rule**
Uniform Deck	NA	53	17
Fixed Deck	27	28	14
Random Deck	NA	36	5

For the further analysis we only used solvers. Table 6 shows that out of 180 solvers in total, 139 (77%) reported a rule. In the Fixed Deck condition roughly the same number of participants reported either the exclusion or the sequence rule.

In a *post-hoc* analysis we compared the solution time of solvers in the Fixed Deck condition, who reported the exclusion rule (*M* = 4.31 min, *SD* = 2.84 min), and solvers, who reported the sequence rule (*M* = 5.77 min, *SD* = 3.59 min). According to a two-sample Wilcoxon test the difference was not significant (*W* = 279, *p* = 0.0975).

In a further *post-hoc* analysis (requested by an anonymous reviewer), we analyzed whether solvers from the Uniform Deck condition who reported a rule showed a different amount of Aha-rating in comparison to solvers who did not report a rule (see Table 7). The underlying assumption was that solutions who were driven by implicit learning were less likely to provide explicit knowledge on the rule and are associated with no Aha experiences.

**Table 7:** Distribution of rules and Aha-ratings in the Uniform Deck condition.

	**Aha**	**No Aha**
Reported rule	44	9
Reported no rule	9	8

The rows are divided into participants who reported the sequence rule or not. The columns show whether the participants had an Aha experience or not.

Table 7 illustrates reporting a rule was stronger associated with an Aha-rating (44/53 = 0.83) than with reporting no rule (9/17 = 0.53). A Fisher exact tests were significant *p* < 0.02.

### Discussion

In Experiment 2 we investigated card sorting as an insight task by introducing a new sorting rule, the sequence rule. In all three conditions the cards could be successfully sorted from left to right, irrespective of the features printed on the face of the cards. In the Uniform Deck condition the deck consisted of only one card, the moon card (see Figure 3), which we assumed was less distracting, because it ruled out the potential application of the standard rules from the beginning. In the Fixed Deck condition, the cards were ordered so that the exclusion rule and the sequence rule could be applied. In the Random Deck condition cards were randomly shuffled. Therefore, the sequence rule sometimes coincided with one of the standard rules, meaning correct feedback could result and be misinterpreted. The observed task difficulty followed our expectations. The Uniform Deck condition was the easiest and the Random Deck condition was the most difficult. Although the solution rate in the Fixed Deck condition was almost the same as in the Uniform Deck condition, the average solution time was more than 2 min higher. In the Fixed Deck condition, solvers found the exclusion rule and the sequence rule with similar probability. The number of solvers, who reported having an Aha-experience, was significantly higher in all three experimental conditions than in the control condition, but there was no significant difference between experimental conditions.

Conceptually, we tried to create conditions that rely on the RCT (see introduction). Finding the solution required problem solvers to overcome the self-imposed constraints that the standard features were part of the solution. Relaxing the prior knowledge constraint was found to be the most difficult in the Random Deck condition. This required a representational change and the search for a new rule. Comparable to our light-switches-problem, it was necessary to consider information, which was not printed on the cards by adding a visual-spatial dimension to the rule space.

This was easiest to attain in the Uniform Deck condition, where the face of the deck cards provided no link to the features of the target cards. The different distribution of Aha and non Aha-ratings for solvers of this condition suggest that the implicit learning assumption could hold true for about half of the solvers who reported no rule. The other half seemed to realize at least a change in behavior, but could not read out the underlying rule. This finding might give us a hint to the nature of the Aha-experience. It seemed at least for our paradigm that deliberate processes can be an important factor for having an Aha-experience. Further investigations would be necessary to clarify this interesting finding and scrutinize the interplay of implicit and deliberate rule induction with the Aha-experience.

## General discussion

### A new card sorting paradigm

In two experiments we pursued the question: Is it possible to convey a well-known card sorting paradigm into an insight-type task? We modified the classical Wisconsin Card Sorting Test in a way that we were able to systematically manipulate the degree of imposed constraints which have to be relaxed in order to solve the problem. After analyzing the found results of the two experiments we positively answered the question with “yes.”

The card sorting game allowed us to add new dimensions, which required that new *features* can be integrated (e.g., letters on condition), *chunks* have to be found (letters below condition), a new rule (exclusion rule) has to be *inferred* (no letters condition) and constraints have to be *relaxed* (uniform deck, random deck condition). We also introduced three ambiguous conditions (letters on, letters below, fixed deck conditions) which can be solved by two alternative rules. Taken together, this provides a large potential of multi-facet insight-type and rule-learning problems.

In our first experiment, we were interested in the question: How was or wasn't additional information on the cards, or below the cards used for a solution? We found that it was easier and more efficient to use the additional letter information when letters were printed on the card in comparison when letters were presented below the cards (chunk decomposition). The solution rate was comparable high in both conditions but problem solvers need more time in the latter condition and some of them use a different and more difficult strategy to solve the problem (26% exclusion rule, 60% letter rule). That is, presenting letters a few millimeters below the cards could initiate a search for a new sorting rule, which did not consider the letter information. The importance of the proper integration of the provided information for the solution process has been proposed and shown for many decades (Köhler, 1925; Duncker, 1945; Grant and Spivey, 2003; Thomas and Lleras, 2007). In general, the basic dimensions (shape, color, number) dominated the search process and imposed a mental set on the search space (Luchins, 1942; Lovett and Anderson, 1996; Öllinger et al., 2008).

In the second experiment, we manipulated other factors that determined the search process. The task was to find a non-obvious sorting rule (sequence rule). This rule required, in analogy to the light-switches-problem, to utilize an additional and non-obvious dimension. In our tasks, participants used the sequential order of cards in the deck. This dimension went beyond the three standard dimensions (shape, color, number). We find that using a deck with one single card that shared no features with the key card resulted in a high solution rate and the fastest solution time. It seemed that having no obvious feature which interfered with the basic dimensions quickly induced the search for new and non-obvious sorting rules.

In contrast, finding the non-obvious sorting rule resulted in lower solution rates when the standard rules were at least sometimes applicable (random deck condition).

In the Fixed Deck condition participants either used the face of the cards as a source of information to infer the exclusion rule, or they sorted the cards based on a visual-spatial sequence rule. Our data suggested that participants had no preference for neither of them.

This was an interesting finding, because it indicated that solvers apply different strategies to solve the task from the beginning. We see the possibility to construe problems which could be solved by different strategies as great advantages of our card sorting game. This might help to respect individual problem solving preferences which could range from deliberate strategies to incidental learning to a combination of both. For example, when searching for a sequence rule, implicit processes could be at work. Those processes facilitated learning via positive feedback (correct mapping) given the contingency between deck card order and target card position (first deck card goes to the left target card, second deck card goes to the second target card from the left).

According to Haider and Frensch (1999), learning the sequence rule could be explained by learning the contingency between the two (see also covariation learning below). As a consequence increasing speed and accuracy can be observed. After these behavioral changes the problem solver deliberately realizes the change and reads out the regularity. Heureka! I found it! It is a sequential rule going from left to right. Please note, the sequence of deck cards and the target sequence could be easily varied. Therefore, our paradigm offers the opportunity to investigate the interrelationship between sequence learning and insight problem solving in much more detail (see Cleeremans et al., 1998 for an introduction).

However, the underlying learning mechanisms remain unclear. Here we provide potential candidate assumptions which might shed light on this important issue.^1^ The first field which is related to our findings is called ordinal position learning. E.g., Terrace (2005) proposed to explain ordinal position learning by the simultaneous chaining theory. His basic idea was that ordinal position learning does not rely on learning chains of stimulus-reaction pairs, but on learning the association of simultaneously presented items and an instructed sequence of these items. This allows the learners to find abstracted rules of item order. In our sequence rule there was an order of the simultaneously presented spatial order of the four target cards and the order of the key cards from the deck. The important point here is that the ordinal position of the target card (from left to right) determined the position of the correct target card and not a certain source card to target card association based on the features on the card. Our results might extend Terrace findings by showing that there might still be an influence of the given features of an item (source cards) which are induced by learning [e.g., contrast between the uniform deck (no inference) and random deck condition (strong inference)].

Covariation learning is another related field which helps to shed more light on potential learning mechanisms. Gaschler et al. (2019), see also Gaschler et al. (2022) and Schuck et al. (2022) investigated how participants used covarying task information. Looking at traffic lights provides color as the main source of information but there is also the position of the lights which provides an alternative source of information. Given this evidence, the authors introduced an analogous task. Participants were asked to respond to the location of large square-shaped stimuli. Additionally, there was color information which covaried with the location of the stimuli in the standard trials. This coincidence was unknown (hidden) to the participants. The authors introduced ambiguous and deviant trials, which varied whether color and/or position determined the response. The authors scrutinized, firstly, whether participants used the color information as a shortcut and secondly, to what extent participants got stuck with the color rule although it was no longer applicable. The data clearly illustrated that participants learned and applied the hidden color rule. However, the amount or use of the rule varied between individuals. Meaning, that some participants relied more on the covariational data than others. This might give us a hint why half of the participants utilized the sequential order of the sequence and the other half utilized the exclusion rule.

In order to evaluate the subjective Aha-ratings in both experiments, we introduced a Aha!-control-condition. The task was to sort a deck of cards which only had letters on the cards. Unexpectedly, 58% of the participants reported an Aha-experience in this simple task. The question arose how our findings could be embedded in the already existing literature. A brief review of recent studies on Aha-experiences showed lower rates of reported Aha-ratings in general. In a series of studies, Danek et al. (2013), Danek and Wiley (2017), and Danek and Salvi (2020) scrutinized the interrelationship of reported Aha! experiences and correct solutions. The authors used classical insight problems and newly invented magic tricks. They reported Aha-ratings between 23 and 70% for magic tricks and about 52% for classical insight problems. In the same vein, Webb et al. (2016, 2018, 2019) investigated different types of problems (classical-insight, CRA, non-insight problems) and their impact on Aha-experiences. The authors found that classical-insight problems elicited Aha-experiences with higher probability than the two other task domains. On average the Aha-rating for classical problems was ~45%. Taken together, all of these findings revealed lower Aha-ratings for classic insight problems than in our control group.

In our reading, the vast difference between the card sorting game and those insight tasks revealed, because the card sorting game required at least 18 consecutive and correct moves to accomplish a valid solution. The existing insight problems often required a single move or needed to integrate an overseen bit of information which determined the solution. Our paradigm drove a more dynamic problem solving process with microsteps and constant trial-by-trial feedback. It remains an open question whether the possibility to report Aha!-experiences during the problem-solving process would change the found pattern.

Currently, we plan further investigations to generalize and test our new paradigm under various conditions. We are very confident that card sorting games will help to shed light on the notion of sequence learning and insight (as proposed by Haider and Frensch, 1999). We also see potential for utilizing the game in neuroscientific investigations. This could help to decipher the neural correlates of insight problem solving going beyond the existing paradigms that often used verbal problems (Jung-Beeman et al., 2004; Luo et al., 2004).

In the next section we provide a first sketch of a potential model which incorporates insight problem solving and card sorting.

### A preliminary model of insight and rule learning

**Figure 5:**
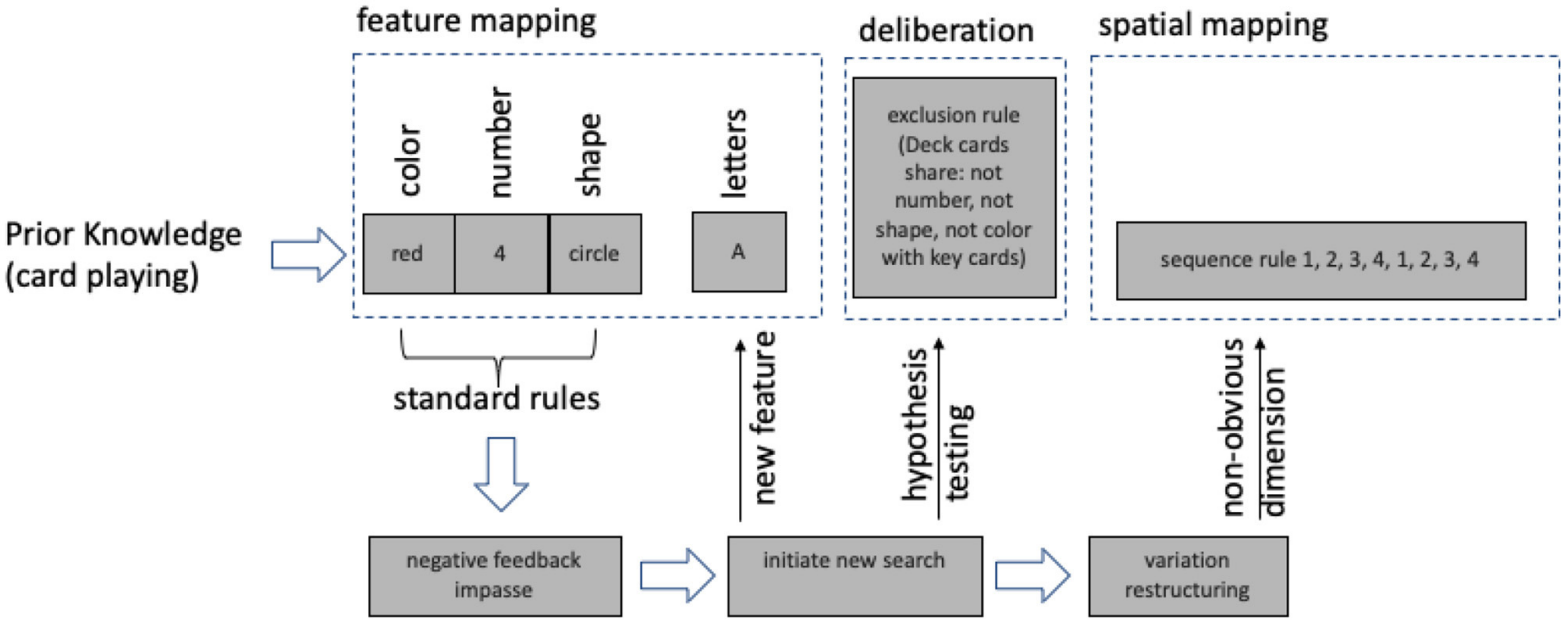
Basic components of our model and information flow.

We assumed that the attempt to embed our findings in an already existing cognitive framework shed light on the underlying computational principles of insight problem solving (see also Hélie and Sun, 2010). Figure 5 provides a flow-chart for a better orientation.

Dehaene and Changeux (1991) proposed a detailed computational model simulating the behavior of the classical WCST. The model relied on an input layer that detected and decoded information which processes form (shape), number and color. Consequently, the model covered the application of the three standard rules (see above). To modify the model for our purpose, a more flexible input layer was required, which enabled the model to decode letter information or incorporate visual-spatial information.

In the authors' model, an important driving factor for switching between rules was negative feedback. Dehaene and Changeux introduced an error cluster and a reward system. For insight problems, negative feedback was often considered as crucial for problem solvers to realize that a solution strategy was not efficient and an impasse was reached (Ohlsson, 1992, 2011; Öllinger et al., 2014). An impasse was supposed to drive unconscious search processes (Seifert et al., 1995; Smith, 1995; Gilhooly, 2016) and to initiate overt search behavior (“initiate new search”) which could help to overcome an impasse (Kaplan and Simon, 1990; MacGregor et al., 2001).

We concluded from our data that there were three different approaches to search for new and insight-type solution strategies. First, in the easiest case new features were added (e.g., letters) to the existing standard rule set—*feature search*. Second, *deliberate search* started to test hypotheses and to infer new rules (such as the exclusion rule). Third, variation of the search criteria provided potential to overcome the standard rule set and enabled the integration of completely new and independent dimensions such as adding spatial information to the rule set—*search by variation*.

We concluded that the high solution rate in the Letters On condition (Experiment 1) reflected the simple addition of letter features as a sorting criterion, especially when the letters were printed on the cards. Here a general rule could be that each type of information, which was printed on the cards, could be used as a sorting criterion. The Dehaene and Changeux model can easily deal with this modification. Constant negative feedback (errors) could result in the search for new features and the extension of the input layer. However, this would require a more flexible input layer that could process letters or other features.

More challenging for the model was the ambiguous results of the Letters Below condition. In this case the model needs an additional mechanism which recognizes and integrates features that were not printed on the cards (letter below the deck card, target card features, feedback). As a consequence it needs a mechanism to build new chunks, namely deck cards and target cards integrate the letter features printed below. This assumption was in accordance with the representational change theory (RCT). For the RCT chunk decomposition of tight chunks (e.g., the cards as an entity) and the composition of new and more flexible chunks played an important role as one mechanism for representational change (see introduction of RCT, Ohlsson, 1992; Knoblich et al., 1999; Öllinger et al., 2006, 2013a).

Furthermore, our data for the Letters Below condition also revealed that some of the participants did not utilize the letters printed below the cards, but started to use the exclusion rule. The exclusion rule could be seen as a logical negation. The rule said to move the deck card to a key card, which shares no single standard feature with it. That is, the given features had to be combined in a way that a completely new rule (exclusion rule) results. For the model this was an interesting situation, because the model needed no additional feature layer, but could rely on the combination of the given standard features and the provided feedback. Consequently, an architecture such as the Dehaene and Changeux model should rather search for the exclusion rule than evolving a new layer which requires a much more flexible architecture. The combinatorial search process could be modeled by a Bayesian hypothesis testing and update process (Griffiths et al., 2008). It was not necessary to discover a new sorting dimension, but to process and to conjunct the given information in a way that the exclusion rule could be inferred. In the next step the model has to test whether the found hypothesis was true by evaluating the feedback. In principle, a negation should be applicable in the Dehaene and Changeux model, which provided a rule coding cluster.

A possible solution for the problem how a new set of rules can be incorporated in the model lies in the combination of the findings of the ACT-R framework (Lovett and Anderson, 1996) and from our own work (Öllinger et al., 2008) and extend it by Bayesian approaches (Griffiths et al., 2008; Chater et al., 2010). Together, the origin of the mental set is explained by the interplay of prior knowledge and the working of a selection-based algorithm. Here a previously successful strategy began to dominate over alternatives in a competitive process. Incidentally, a similar approach was at the heart of Bayesian inference (Griffiths et al., 2008; Chater et al., 2010). It was shown that the fundamental equation of evidence-based inference is isomorphic to the discrete-time replicator equation (Harper, 2009). This raised the questions to what extent Bayesian-type cognitive processes might mechanistically be realized by within-brain bona fide selection and evolution (Suchow et al., 2017). In our reading this insight could be understood as the interplay of hypothesis testing and the start of variation, if the first did not provide further progress.

In this vein, we showed that evolutionary dynamics successfully solved a simplified version of the four-tree problem (Fedor et al., 2017). The problem states:

“A landscape gardener is given instructions to plant four special trees so that each one is exactly the same distance from each of the others. How is he able to do it?” (De Bono, 1971).

Most of the participants began to search in 2D space. The problem required searching for the solution within a 3D search space. The solution was a tetrahedron. The model demonstrated how Baysian inference and variation driven by evolutionary processes played together and modeled phases of prior knowledge hypothesis testing and evolutionary variation, when no further progress was possible, induced a representational change. This approach was in line with existing evolutionary accounts addressing insight and creativity (Campbell, 1960; Simonton, 2011), but goes beyond it, because the generation of models (replication) relied on the already learned information and the best candidate solutions, which were tested against a fitness function. Dietrich and Haider (2014) and Dietrich (2015) provided a similar account for creative problem solving which relied on offline simulations of promising motor outcomes which were scaffolded by the goal representation and prior progress. Until now, models such as ACT-R or the Dehaene and Changeux model entailed, at least to our knowledge, no mechanism that combines variation and learning of new rules or dimensions.

Both finding the spatial sequence in our modified task and discovering the warmth information in the light-switches-problem crucially depended on the variation of the problem representation to extend the search space for new candidate solutions (Öllinger and von Müller, 2017). Finding the spatial information in our paradigm could be seen analogous to finding a 3D representation in the four trees problem. In the card sorting game candidate solutions could be validated by the provided feedback of the card sorting game (fitness criterion). Again, after establishing a new search dimension (e.g., spatial information), reinforcement learning rewarded the spatial information and the sequence could be learned. As a result, the new rule could be learned and integrated in the rule repertoire of the model.

It remains to be seen how far this cognitive approach of rule evolution can be generalized for any type of rule learning, including a model for the findings reported here.

Finally, let's switch to a completely other field outside the realm of human cognition in order to speculate about the generalizability of the interplay of prior knowledge modification and variation for problem solving. It was shown that clear cases of representational change even were found in the realm of evolutionary biology. A fascinating example was the numerical simulation of a population of RNA molecules evolving in different environments (Parter et al., 2008). To cut it short, different environments favor (select for) different secondary RNA structures. It was found that it takes a shorter time for the population to re-adapt (by mutation and selection) to a previously already experienced environment (after having evolved in a different one) than the time it took for the naive population to evolve in the first place. The reason is that the population accumulated certain positions in the primary structure (the sequence) which, when mutated, resulted in a radical restructuring of the secondary structure (the phenotype). These switches were lacking in the naive population. The evolved population was even able to generalize to unseen environments, provided the target structure belonged to the same grammatical class with the structures favored in the training environments. This analogy raised the following possibilities for our topic: (i) alternative candidate solutions might be encoded in the form of dynamical attractors which are maintained simultaneously (ii) in competition with each other (iii) so that better solutions will dominate that (iv) can act as mental blocks in the case of new challenges, (v) unless the nature of attractors is such that, when perturbed, flip readily into an alternative state encoding a potentially useful, restructured representation. A prediction is that the representations in subjects, who are better at insight problem solving, are such that they allow for facilitated variation. How this can be neuronally encoded is a most exciting question (Fernando et al., 2008, 2010, 2012; Hélie and Sun, 2010; Sun, 2016).

## Data availability statement

The datasets presented in this study can be found in online repositories. The names of the repository/repositories and accession number(s) can be found below: OSFHome: https://osf.io/w9sbe/?view_only=d165197f4f86448d98f00e6feb93c943.

## Ethics statement

Ethical review and approval was not required for the study on human participants in accordance with the local legislation and institutional requirements. The patients/participants provided their written informed consent to participate in this study.

## Author contributions

MÖ and AF planned the study and collected the data. AF analyzed the data. MÖ wrote the manuscript. ES provided the evolutionary and modeling background. All authors contributed to the article and approved the submitted version.

## Footnotes

^1^We would like to take the opportunity to thank an anonymous reviewer who brought these points to our attention.
