# Supplementary material for: Search and insight processes in card sorting games
Source: Front Psychol. 2023 May 5;14:1118976. doi: 10.3389/fpsyg.2023.1118976 (PMC10196050; doi:10.3389/fpsyg.2023.1118976)
Supplement: Supplementary file 1 [file Data_Sheet_1.PDF]

Appendix 1. The Deck

The deck consisted of these cards in random order in all three experimental conditions of Experiment 1: In the Letters On condition the letters were displayed on the cards, at the bottom, in the Letters Below condition the letters were displayed below the cards and in the No Letters condition, the letters were not visible. The same deck was used in the Random Deck condition of Experiment 2 (without letters), randomly shuffled. In the Fixed Deck condition of Experiment 2, the same deck was used, but the cards in the deck were in the exact same order as they are listed here.

**Order of cards**	**Card dimensions/features**				**Correct placement according to the**			
	**Number**	**Color**	**Shape**	**Letter**	**Number rule**	**Color rule**	**Shape rule**	**Letter rule**
1	four	yellow	star	A	D	C	B	A
2	one	blue	rectangle	B	A	D	C	B
3	two	red	circle	C	B	A	D	C
4	three	green	triangle	D	C	B	A	D
5	two	blue	rectangle	A	B	D	C	A
6	three	red	circle	B	C	A	D	B
7	four	green	triangle	C	D	B	A	C
8	one	yellow	star	D	A	C	B	D
9	four	green	rectangle	A	D	B	C	A
10	one	yellow	circle	B	A	C	D	B
11	two	blue	triangle	C	B	D	A	C
12	three	red	star	D	C	A	B	D

13	two	yellow	circle	A	B	C	D	A
14	three	blue	triangle	B	C	D	A	B
15	four	red	star	C	D	A	B	C
16	one	green	rectangle	D	A	B	C	D
17	three	blue	star	A	C	D	B	A
18	four	red	rectangle	B	D	A	C	B
19	one	green	circle	C	A	B	D	C
20	two	yellow	triangle	D	B	C	A	D
21	three	green	circle	A	C	B	D	A
22	four	yellow	triangle	B	D	C	A	B
23	one	blue	star	C	A	D	B	C
24	two	red	rectangle	D	B	A	C	D

Appendix 2. Settings for sample size calculations in G*Power

We used G*Power 3 for the analysis (Faul et al. 2007).

Test family	Exact
Statistical test	Proportions: inequality, two independent groups (Fisher's exact test)
Type of power analysis	A priori: Compute required sample size - given α, power and effect size

Tail(s)	Two
Proportion p1	1
Proportion p2	0.9
α error probability	0.05
Power (1-β err prob)	0.8
Allocation ratio (N2/N1)	1

Since these are new tasks, we could only guess the proportion of different outcomes (solving or not solving the task) in the different conditions. For Experiment 1 we predicted that the solution rate will be 100% in the Letters On condition (p1) and maximum 90% in the Letters Below condition (p2). These parameters result in a sample size of 78. For the No Letters condition we predicted a 20% solution rate (p1 = 90%, p2 = 0.2), which results in a sample size of 9.

For Experiment 2, we predicted 0.8, 0.3 and 0.1 solution rates. All these settings resulted in smaller sample sizes, but we decided to go with a sample size of 78 per condition in Experiment 2 too. The reason for this is that for the Aha-ratings analysis we used a common control condition across Experiment 1 and Experiment 2.
